# Effects and molecular mechanism of endophytic elicitors on the accumulation of secondary metabolites in medicinal plants

**DOI:** 10.3389/fmicb.2025.1558567

**Published:** 2025-06-02

**Authors:** Yaxuan Wang, Nana Chen, Kuanping Deng, Xinying Zhong, Zhaogao Li, Lin Li, Delin Xu

**Affiliations:** ^1^Department of Cell Biology, Zunyi Medical University, Zunyi, China; ^2^Zunyi Academy of Agricultural Sciences, Zunyi, China; ^3^Department of Medical Instrumental Analysis, Zunyi Medical University, Zunyi, China

**Keywords:** endophytic, secondary metabolite, biosynthesis, biological mechanism, medicinal plant

## Abstract

Endophytes in medicinal plants possess significant biological value since they have in them the ability to provide elicitors crucial in regulating plant growth as also different secondary metabolites. This review emphasizes the effective ability of endophytic fungi to induce their hosts, explains the biological mechanisms of using endophytic fungi to enhance the accumulation of secondary metabolites in medicinal plant cultures, and summarizes the extensive application of endophytic fungi elicitors in medicinal plant cultivation. The main goal of this article is to clarify the mechanism and important role of endophytic elicitors in the production of natural drugs. This information will be helpful for scientific researchers in controlling the quality of medicinal materials, prioritizing endophytic resources, and achieving a circular and sustainable development and production of natural medicine.

## Highlights

- Endophytic elicitors significantly boost secondary metabolite levels in medicinal plants.
- Elucidates molecular pathways involving JA, SA, and ROS in plant-endophyte interactions.
- Advances in metagenomics and bioinformatics reveal diverse endophyte communities.
- Enhances sustainable cultivation of medicinal plants through microbial symbiosis.
- Highlights potential biotechnological applications of endophytic fungi elicitors.

## 1 Introduction

The use of natural drugs for treating or preventing diseases has been the primary means of safeguarding human health for thousands of years. The further exploitation of medicinal components found in plants is currently a focal point in drug development. However, due to prolonged land and forest degradation caused by human activity, the availability of natural medicinal resources has dwindled. Rare medicinal plants often exhibit characteristics such as limited natural distribution, weak regenerative capabilities, extended growth cycles, and challenging chemical synthesis of active ingredients. As a result, the pharmaceutical industry reliant on plant-derived materials has become resource intensive, leading to a significant surge in the price of traditional Chinese medicinal materials plant in the market. The challenge, therefore, lies in striking a balance between resource protection and sustainable utilization within the pharmaceutical industry. Extracting more effective secondary metabolites from endophytic fungi and host plants' symbiotic systems has gradually become a new method for conserving medicinal natural resources and reducing resource wastage.

Digging out endophytes from medicinal plants to synthesize medicinal ingredients or their precursors provides a new approach for the protection of medicinal plants. Previous studies have shown that the interaction between endophytic fungi and hosts is different from the process of pathogenic fungal infection. The interaction between endophytic fungi and hosts is a balance and long-term coevolution between virulence factors of endophytic fungi and host defense responses, gradually forming a relatively stable equilibrium for long-term coexistence (Wang et al., 2021). However, in reality, the mechanisms maintaining this equilibrium are far more complex and intricate than the balance itself (Cheng et al., 2024). In the symbiotic system of endophytes and host plants in a stable equilibrium, endophytes can regulate host plants through three ways. These include direct synthesis of metabolites (Lv et al., 2024), selective catalytic synthesis of metabolites (bioconversion) (Tian et al., 2014), and induction of a secondary metabolite effect or horizontal gene transfer (Ku et al., 2024; Ambrose et al., 2014). Among them, relevant studies have shown that endophytic fungi isolated from *Camptotheca acuminata* can directly biosynthesize camptothecin. This finding demonstrates that endophytic fungi can maintain a stable symbiotic system through direct synthesis of metabolites (Kusari et al., 2011). In addition, researchers monitored changes in leaf compounds of *Cephalotaxus harringtonia* caused by the endophytic fungus *Paraconiothyrium variabile* using metabolomics methods, and then characterized the altered products. They observed the specific biotransformation of glycosylated flavonoids by the endophytic fungus (Tian et al., 2014). These studies provide strong evidence for the important role of endophytic fungi in regulating the stability of symbiotic systems. Based on genomics, metabolomics, and microbiomics, we can discover that endophytic fungi act as special “inducers” to regulate the growth metabolism of plants in the plant-microbe interaction system (Ding et al., 2022), In addition, endophytic fungi enter plant tissues and alleviate biotic and abiotic stress by producing a large number of secondary metabolites (Li et al., 2022). They are dedicated to *de novo* synthesis of structural compounds and stimulate plant immunity (Yipare et al., 2022). They also participate in the process of excluding pathogens through niche competition and actively participate in the phenylpropanoid metabolism process. In addition, we also pay attention to the fact that endophyte metabolites have efficient growth-promoting and regulatory effects on host cells *in vitro* (Wang et al., 2022; Tran et al., 2022; Agarwal et al., 2020). Therefore, the development of efficient inducers in plant tissue culture technology, elucidation of the mechanisms by which endophytic fungi directly synthesize or induce plants to synthesize effective components, are of special significance in solving the current dilemma of medicinal plant production and resource conservation, and also promotes the sustainable production of secondary metabolites from medicinal plants.

## 2 Endophytic elicitors possess the power to effortlessly coax the synthesis of valuable medicinal components

**Figure 1:**
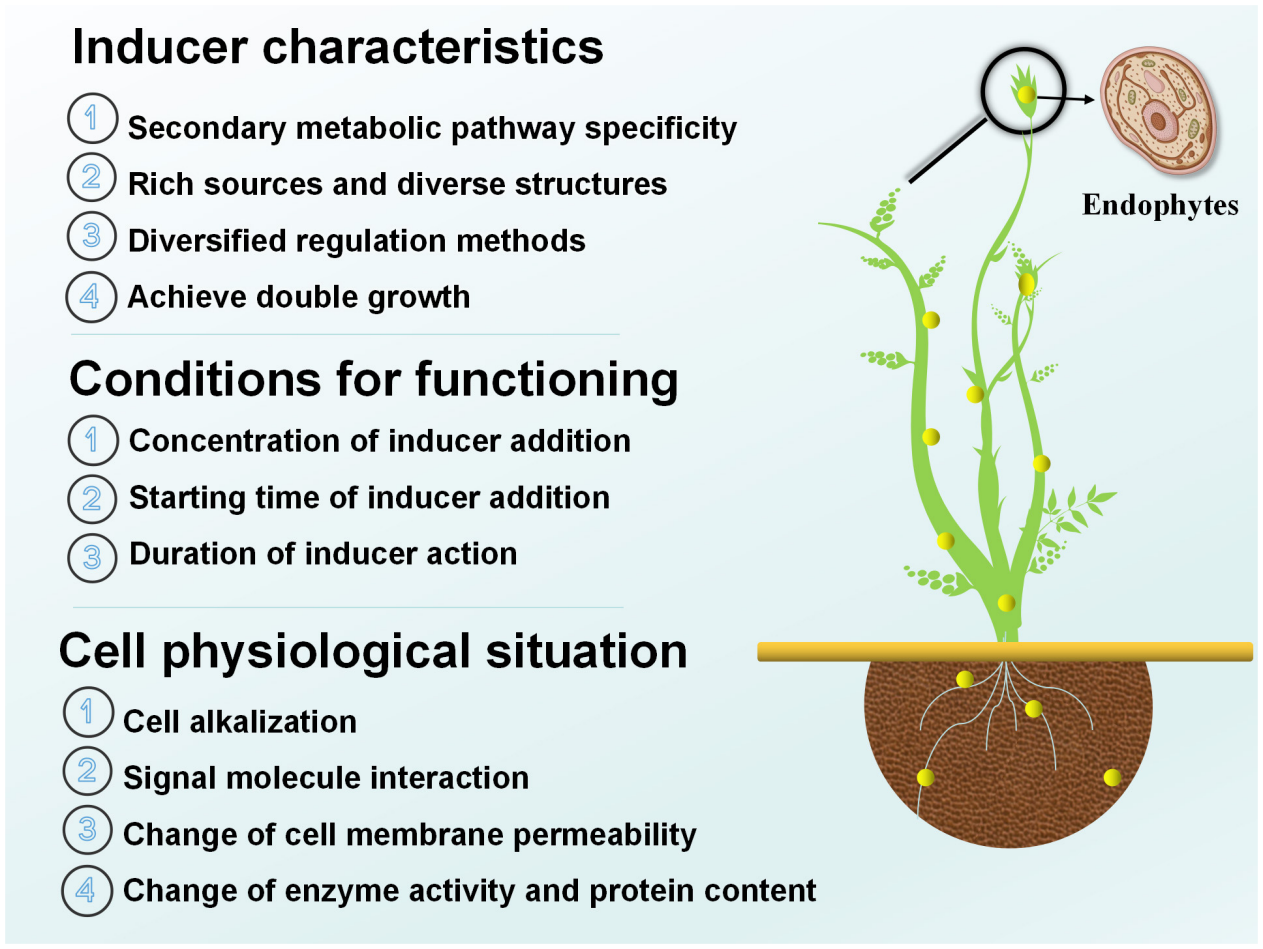
Characterization, conditions, and effects of efficient induction of endophytes on the physiological posture of host cells.

Endophytic elicitors are external biological elicitors, whose use to control the production of active components in medicinal plants has gained popularity in research. By considering factors such as the unique characteristics of different inducers, the conditions required for induction, and their impact on the physiological state of host cells (Figure 1), The rational selection and effective implementation of inducers can promote the accumulation of secondary metabolites in medicinal plants (Zhang, 2022).

### 2.1 The fascinating characteristics of endophytic inducers

Endophytic elicitors have significant advantages over abiotic elicitors and pathogens in regulating the synthesis of secondary metabolites in the host due to their unique properties.

**Figure 2:**
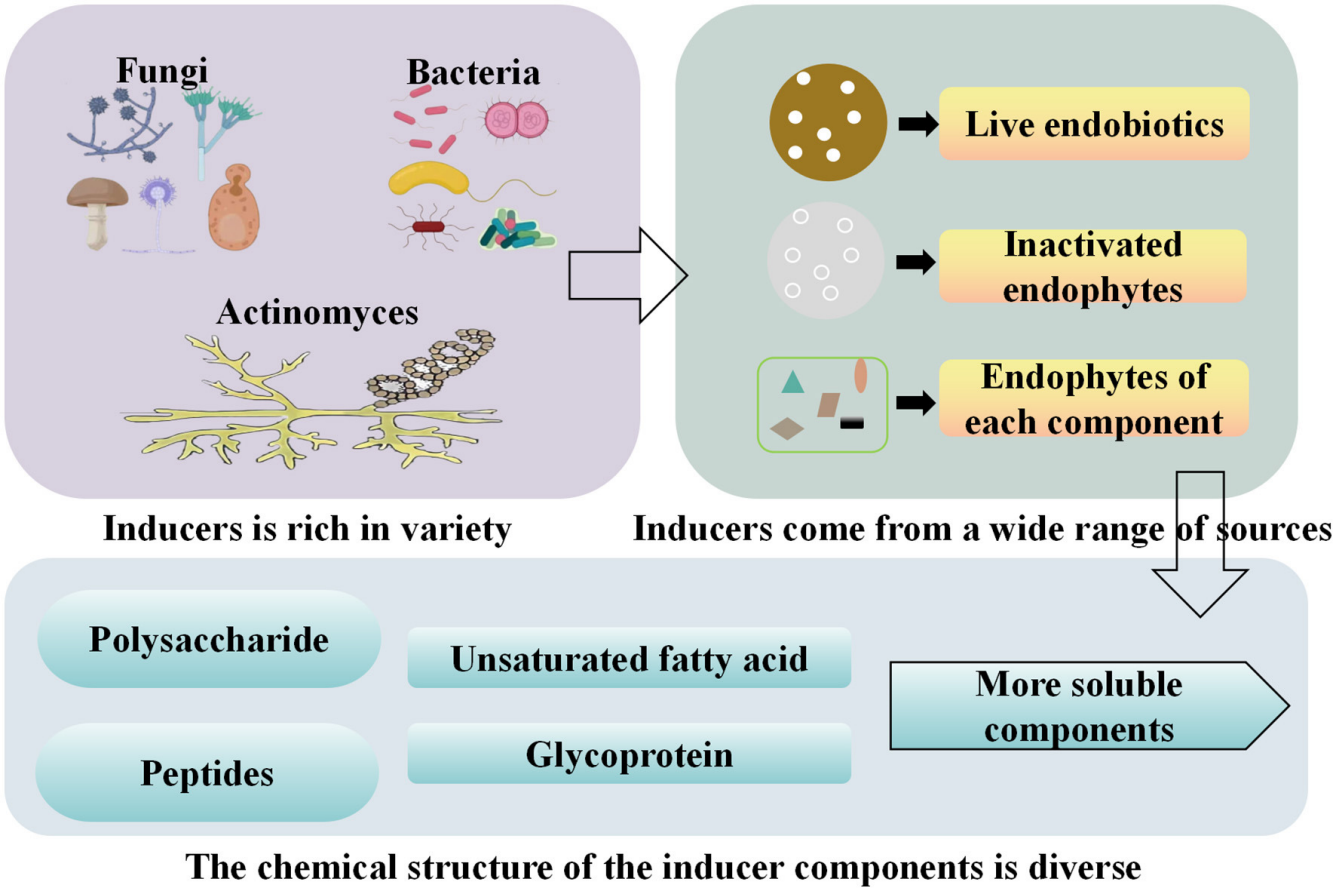
Classification, source and active components of endophytic inducers.

These inducers have a diverse range of sources and structures (Figure 2). The endophytes within the symbiotic system exhibit a rich variety of species, including endophytic bacteria, fungi, actinomycetes, and yeasts. The key components of the induction mechanism are mainly derived from endophytic viable cells, endophytic inactivated cells, and components of endophytic bacteria. These chemical structures include polysaccharides, polypeptides, glycoproteins, and unsaturated fatty acids.

There are various methods to regulate inducers. Current research has discovered that endophytic fungi can specifically regulate the production of secondary metabolites by inducing the synthesis of new enzymes and increasing the content of key enzymes in primary metabolic pathways (Cao et al., 2024).

This leads to high induction efficiency and dual growth, meaning that gentle stimulation from endophytic elicitors can avoid plant destruction caused by pathogenic elicitors and promotes the simultaneous growth of host cells and secondary metabolites (Liu et al., 2021).

The activation of secondary metabolic pathways by elicitors in medicinal plants is specific to species and growth stages. Specific metabolic pathways contribute to the efficient accumulation of particular secondary metabolites (Singh et al., 2023). Therefore, it is necessary to establish an optimal induction culture system and strictly control induction conditions based on the characteristics of the inducer.

### 2.2 Action conditions of endophytic elicitors

The effectiveness of endophytic inducers is closely related to various factors, including the concentration, of the inducer, the timing of its addition, and the duration of its action. The concentration effects of endophytic elicitors can be classified into two types: reactive saturation type and optimal concentration type. In general, excessive inducers can cause plants to have an excessive immune response, resulting in the production of toxin substances that damage the host plant cells and cause localized cell death. Suitable concentrations of fungal inducers can induce the synthesis and accumulation of active substances that stabilize products in the interaction between plants and microorganisms when the HR resistance reaction occurs (Zhu et al., 2024). In most medicinal plant cell cultures, the accumulation of secondary metabolites reaches its peak after the exponential growth period, so it is important to select an appropriate time for the addition of inducers.

**Figure 3:**
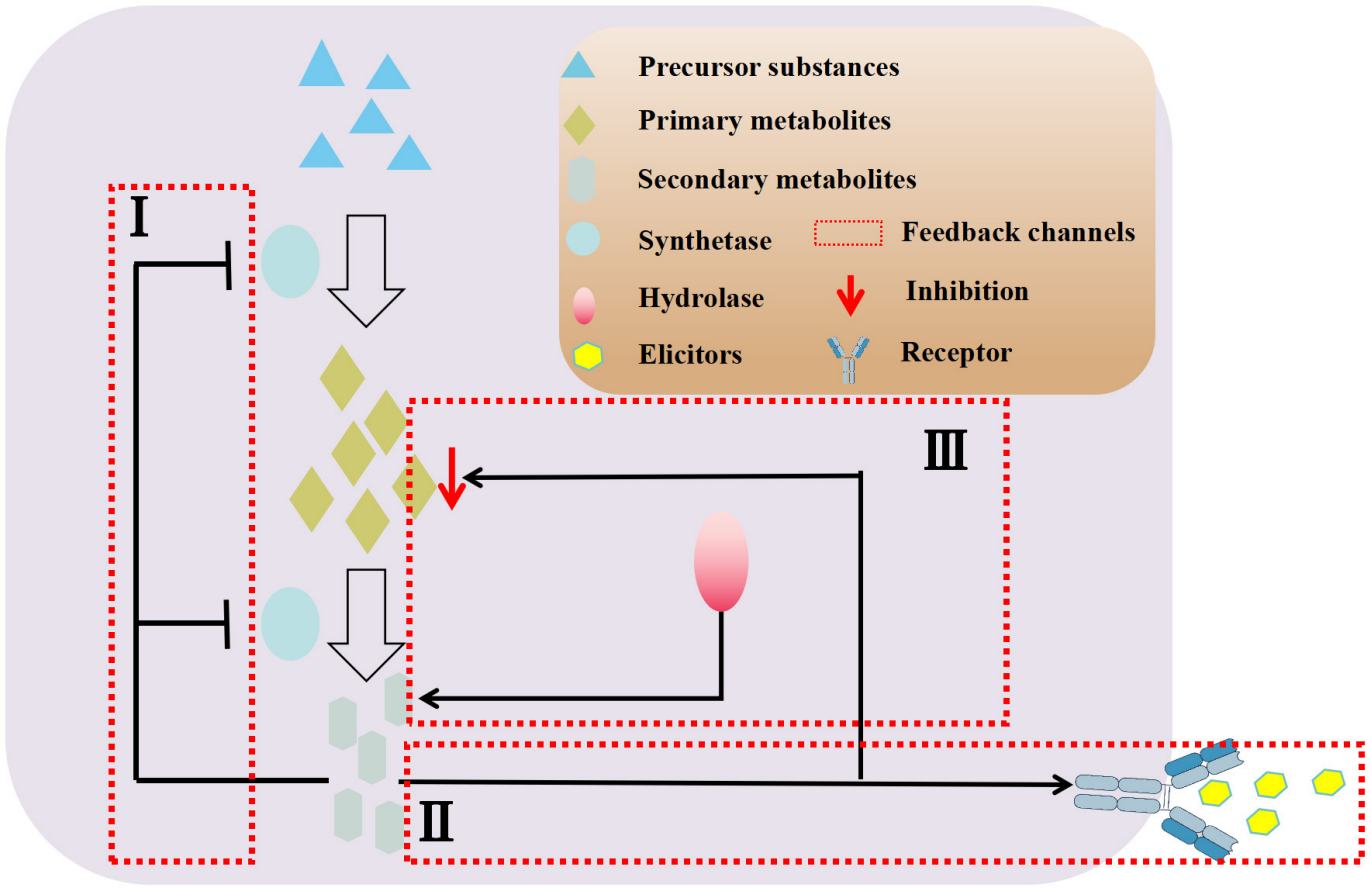
Negative feedback regulation mechanisms of secondary metabolites in co-culture system.

Furthermore, adding the correct amount of inducers at the optimal time does not guarantee the maximum amount of secondary metabolites (Wang et al., 2004). Over time, some plant culture systems may experience a decline in the production of secondary metabolites. This can be attributed to factors such as the excess of synthesized secondary metabolites inhibiting the synthesis pathway through feedback, competition between secondary metabolites and endophytic inducers for binding sites in plant cells (Figure 3), and a lack of raw materials for primary metabolites (Pang et al., 2021).

### 2.3 Effects of endophytic elicitors on the physiological situation of medicinal plant cells

When endophytic fungi induce derivative to join the plant cell suspension culture medium, the plant undergoes changes in cell membrane permeability, enzyme activity and protein content, cell alkalization, and signaling molecule interaction in order to protect itself, thereby affecting the accumulation process of secondary metabolites (Wróbel-Kwiatkowska et al., 2024). After the endophytic inducer binds to receptors on the cell membrane, it causes an increase in membrane permeability and changes in fluidity, leading to cell infiltration. At the same time, it can cause metabolic changes in cells, enabling them to actively synthesize phytoalexins, lignin, etc., required for plant defense, strengthening specific secondary metabolic pathways (Li et al., 2024; Liu Y. H. et al., 2024). The occurrence of these processes is mainly achieved by regulating the amount and activity of enzymes, and changes in protein content reflect the quantitative change process of these enzymes. For example, after adding *Aspergillus oryzae* inducer on the 8^th^ day of cell culture of *Arnebia yunnanensis*, the content of intracellular soluble protein increases rapidly (Ning et al., 1996).

In addition, endophytic inducers also induce the alkalization of the cell culture system. The researchers found that before induction, the *Taxus* cells were in a relatively stable growth state, with intact cells and well-developed organelles. The redox potential was low, and the enzyme system related to reactive oxygen species (ROS) was relatively stable. The primary metabolism of the cells was vigorous, but the synthesis rate of paclitaxel was low. After induction, the *Taxus* cells shifted toward product synthesis. The activity of superoxide dismutase (SOD) increased rapidly, while the activities of catalase (CAT) and peroxidase (POD) were strongly inhibited. ROS burst occurred in the cells, and the redox potential increased. Ion channels on the cell membrane were activated, leading to the influx of H^+^ and Ca^2+^ and the efflux of Cl^−^. The vacuoles showed a large number of high-electron-density areas containing Ca^2+^ ions, exhibiting regular changes. The culture environment became alkaline, inhibiting cell growth but increasing the synthesis rate and yield of paclitaxel (Zhang et al., 2001). The synergistic activation of the intracellular phospholipase C dual messenger pathway and Ca^2+^ channel can accelerate the accumulation of inducers for paclitaxel synthesis, while Ca^2+^ influx significantly enhances the signal of intracellular oxygen burst (Li et al., 2002). Oxygen bursts and nitric oxide (NO) induced by endophytic elicitors have become key components in the signal transduction network of secondary metabolism in medicinal plant cells. These elements can work in conjunction with signal molecules like reactive oxygen species (ROS), jasmonic acid (JA), and salicylic acid (SA) to jointly regulate the biosynthesis of secondary metabolites in plant cells (Lv et al., 2021).

### 2.4 Screening of endophytic inducers

The effective selection of inducers is based on the advantage that endophytic inducers can fully utilize their highly effective induction effects, by selectively inducing specific secondary metabolites through the selection of suitable endophytic bacteria or endophytic components. For instance, fungal elicitors can rapidly activate phenylalanine ammonia lyases in the hairy roots of *Loiuscor niculatus* to synthesize isoflavones, without significantly affecting other unrelated enzymes. Jian and colleagues conducted a screening of various elicitors from 12 fungal substances to treat suspension-cultured cells of *Catharanthus roseus*, and the results showed that different mycelium homogenates could stimulate production of diverse indole alkaloids (Zhao et al., 2001). A large number of studies have demonstrated the efficient inducing effect of endophytic fungi. However, in order to achieve effective screening and fully utilize the induction effect, it is necessary to must first understand the synthesis pathway of specific secondary metabolites, the structure and characteristics of key enzymes, and the relationship between the structure and function of inducers and secondary metabolites. Therefore, elucidating the mechanism of endophyte inducers is not only essential for optimizing screening strategies but also crucial for uncovering the co-evolutionary relationships between plants and microbes. Effective screening of endophyte inducers will significantly improve the efficiency of targeted synthesis of secondary metabolites in medicinal plant cells and offer a new paradigm for the development of natural drugs.

## 3 Delving into the mechanism of endophytic elicitors

**Figure 4:**
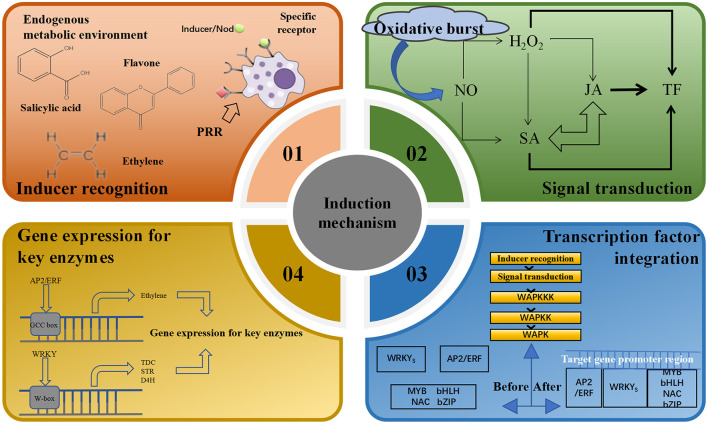
Induction mechanisms of endophytic elicitors.

Endogenous inducers can affect physiological and biochemical levels, gene expression and metabolic pathways in host cells as they induce the accumulation of secondary metabolites in plants. Over time, the collaborative interaction between the two results in a comprehensive and complex induction mechanism (Cao L. S. et al., 2022; Bauer et al., 2001). Recognition of the source of endophyte inducer stimulation by the host plant body is central to the plant's ability to respond to cellular changes such as activation of kinases, production of reactive oxygen species, ion fluxes and cytoplasmic acidification. Receptors on the surface of the plasma membrane of plant cells can recognize different structures of specific inducers, trigger the plant to generate a signal transduction network, elicit a plant response, and transmit signals to transcription factors under stress (Wu et al., 2024). Changes in the mRNA transcript levels of the relevant genes trigger changes in the expression of key enzymes involved in secondary metabolites, regulating cellular metabolism and leading to the accumulation of certain secondary metabolites (Zhang et al., 2022; Vasconsuelo and Boland, 2007; Yu and Facchini, 2000), thus regulating the immune defense system of the host plant (Bu et al., 2023). For example, treatment of poppy cell suspension cultures with fungal triggers induces glutathione s-transferase (GST) synthesis, which catalyzes a GSH-coupled reaction and regulates the levels of secondary metabolites. Thus, the complete molecular mechanism of action of elicitors on secondary metabolism consists of four levels: inducer recognition, signal transduction, transcription factor integration, gene expression and activation of key enzymes (Figure 4).

### 3.1 Inducer identification

In numerous studies on the effects of endophytes on the accumulation of effective components in plants, it has been discovered that the initial step in establishing symbiotic relationships (Qu et al., 2021; Tian and Nan, 2017) and inducing the synthesis of secondary metabolites is the recognition of endophytic elicitors by host cells. The characteristics and genetic composition of microbial communities constructed by different plants in the same habitat gradually change over long-term evolution (Ehinger et al., 2009). Therefore, symbiotic systems jointly determine whether to initiate induction programs based on the metabolic environment and multiple receptors in the habitat (Limpens et al., 2003; Thoms et al., 2021). In complex metabolic environments, some substances play particularly important roles. These include nutrients that are absorbed and utilized by endogenous bacteria, antibacterial substances that can selectively eliminate toxic pathogens, and specific compounds that attract specific endogenous bacteria (Ancheeva et al., 2020). For example, the roots of *Arabidopsis thaliana* secrete organic acids that serve as important nutrients for recruiting *Bacillus subtilis* (Rudrappa et al., 2008). The inoculation of *Pseudomonas fluorescens* improved the plant nutrient cycle and enhanced the adaptation of the interleaf microbes to the host environmental conditions, suggesting that the ability to utilize host nutrients may be a key factor in symbiotic association (Li et al., 2025; Choi and Klessig, 2016; Liu et al., 2018).

Similarly, plants may create a wide spectrum of antimicrobial chemicals, but the regulation mechanisms of how these molecules resist pathogenic bacteria while allowing endophytes to grow have not been fully explained. However, experiments have fully demonstrated that plants can use metabolites to selectively choose specific endophytes while excluding other microorganisms. For example, coumarins derived from plants have antibacterial activity against some pathogenic bacteria, but do not exhibit antibacterial activity against endophytic bacteria (Voges et al., 2019).

Moreover, metabolic components such as flavonoids, benzoxazines, and ethylene can all act as specific attractants for specific endophytes (Ran et al., 2024; Harbort et al., 2020). The current research on these metabolic compounds provides a basis for plants to selectively utilize advantageous endophytes in complex microbial communities. The specific metabolic environment of the symbiotic system has the potential to become the primary determining factor in the formation of specific symbioses between host plants and endophytes.

When under the attack from host antibacterial metabolites, endophytic actively compete for nutrients and establish symbiotic relationship with host plants when they successfully survive and reproduce. During this process, endophytes engage the induction mechanism; nevertheless, activation of the induction mechanism requires many receptors to sense and integrate complex and diverse signals. Although some inducer affinity proteins encoded by the pattern recognition receptor (PRR) in plant genomes have been successfully isolated (Zhang et al., 2010), further research is needed to study the structure and function of related receptor proteins on the cell membrane of medicinal plants. In addition, the endogenous fungal effector NOd can also act as a ligand receptor to participate in the recognition of inducers (Sun et al., 2022; Miyata et al., 2016). For example, there are two assumed Nod factor receptors, NFP and LYK3, that can recognize inducers of Nod factors in the case of clover and alfalfa. Among them, MtNFP acts as the signal receptor for Nod factors, and MtLYK3 acts as the entry receptor for Nod factors. In the dual receptor model, both of them jointly regulate the downstream signaling pathway (Liu et al., 2023; Jones et al., 2007). Additionally, the uptake of endophyte metabolites and hormones by host cells also creates conditions to promote the occurrence of endophyte-induced secondary metabolism and the establishment of symbiotic relationships (Etalo et al., 2018; Furtado et al., 2019; Xia et al., 2022; Chen et al., 2023).

Endosymbiotic microorganisms that establish a symbiotic relationship with the host plant exhibit gene expression that is largely distinct from that of other microorganisms and possess the potential to produce novel secondary metabolites (Wang L. et al., 2024). Metabolites produced by endophytic microorganisms may also regulate the synthesis of more diverse metabolites by the host plant (Raza et al., 2020; Schenkel et al., 2019), favoring host plant growth while influencing microbial colonization (Shen et al., 2015; Johri et al., 2015; Hong et al., 2024). Therefore, different receptor molecules located on the plasma membrane of the host plant cell can recognize and bind specific endogenous enzymes. This process has certain selectivity. Subsequently, symbiotic signals are transmitted to downstream intracellular signaling molecules (Mengistu, 2020; Santoyo, 2022).

### 3.2 Signal transduction

Intracellular signal transduction is a crucial link between endophytic elicitors and the secondary metabolites of medicinal plants. Once the host plant detects the endophytic inducer, the cell membrane depolarizes, resulting in the opening and closing of ion channels and the coupling of G proteins. This activation triggers various messenger systems that enhance the induction signal (Zhao et al., 2023). Research and summaries of different medicinal plants such as *Artemisia annua, Atractylodes macrocephala, Ginkgo biloba, Pueraria lobata, Astragalus membranaceus*, and *Taxus chinensis*, have revealed that NO, ROS, JAs, and SA are key signaling molecules that regulate the growth, development, expression of resistance genes, and accumulation of pharmacologically active substances in host plant cells induced by endophytic bacteria in medicinal plants (Zhang et al., 2015; Qi et al., 2021).

NO serves as an important upstream signaling molecule in the regulatory network. It can mediate the process of endophytic elicitors inducing the synthesis of secondary metabolites in plants through two different pathways, partially depending on oxidative burst and partially independent of oxidative burst. For instance, the exogenous addition of NO can improve PEPC enzyme activity and promote the generation of cyclopheneether terpenoids in gentian (Song et al., 2023). Additionally, applying NO alone can promote the production of ROS in *Taxus chinensis* cells (Xu and Dong, 2006a). This suggests that the accumulation of ROS synthesis in the endophytic induction mechanism is a downstream signal transduction event of the NO pathway. Among different host plant cells, the occurrence of H_2_O_2_ is the most probable in oxidative bursts (Jedelská et al., 2021; Zheng et al., 2016). For example, H_2_O_2_ appears in the induction of β-eucalyptol synthesis in *Atractylodes lancea* cells (Cao L. S. et al., 2022).

However, NO enhances the synthesis of secondary metabolites while inhibiting the accumulation of most ROS in the cell. Studies have shown that specific NO specific scavengers (cPTIO) and lipoxygenase inhibitors (NDGA) can hinder the promotion of NO and JA on the synthesis of plant secondary metabolites in the cell wall inducer pathway of *Aspergillus niger* (Ren, 2014). Furthermore, experimental results demonstrate that increasing intracellular JA content in hypericum cells treated with NO alone indicates that NO is located upstream of JA and has a promoting effect on JA accumulation (Song et al., 2023).

JAs also plays a significant role in signal transduction pathways that promote plant growth, enhance plant resistance (Chen et al., 2020; Mao et al., 2021), and stimulate the synthesis of plant secondary metabolites (Wang X. Y. et al., 2024). Yan et al. (2019) discovered that the content of MeJA and volatile oil increases when the inducer of *Penicillium* endophytic fungus 2J1 induces *Cinnamomum longepaniculatum* (Gamble) N. Chao suspension cells. Other studies have confirmed that both JA and SA are signal molecules involved in PB90 (a protein elicitor from *Phytophthora boehmeriae*) induced flavonol glycoside production, and there is a specific interaction between the two (Ullah et al., 2022; Xu and Dong, 2006b). SA can bind to various SABP binding proteins, suggesting that it can mediate different pathways of induced responses in host cells. For example, SA can effectively counteract the damaging effect of excessive ROS on functional molecules (Liu A. et al., 2024), and it can also directly mediate the accumulation of pilocarpine in pilocarpine (Chen et al., 2021b). The endophytic *Acinetobacter sp*. ALEB16 induces and stimulates medicinal plant *Atractylodes lanceolata* to synthesize SA, accelerating the accumulation of volatile oils from *Atractylodes lanceolata* (Wang et al., 2015).

**Figure 5:**
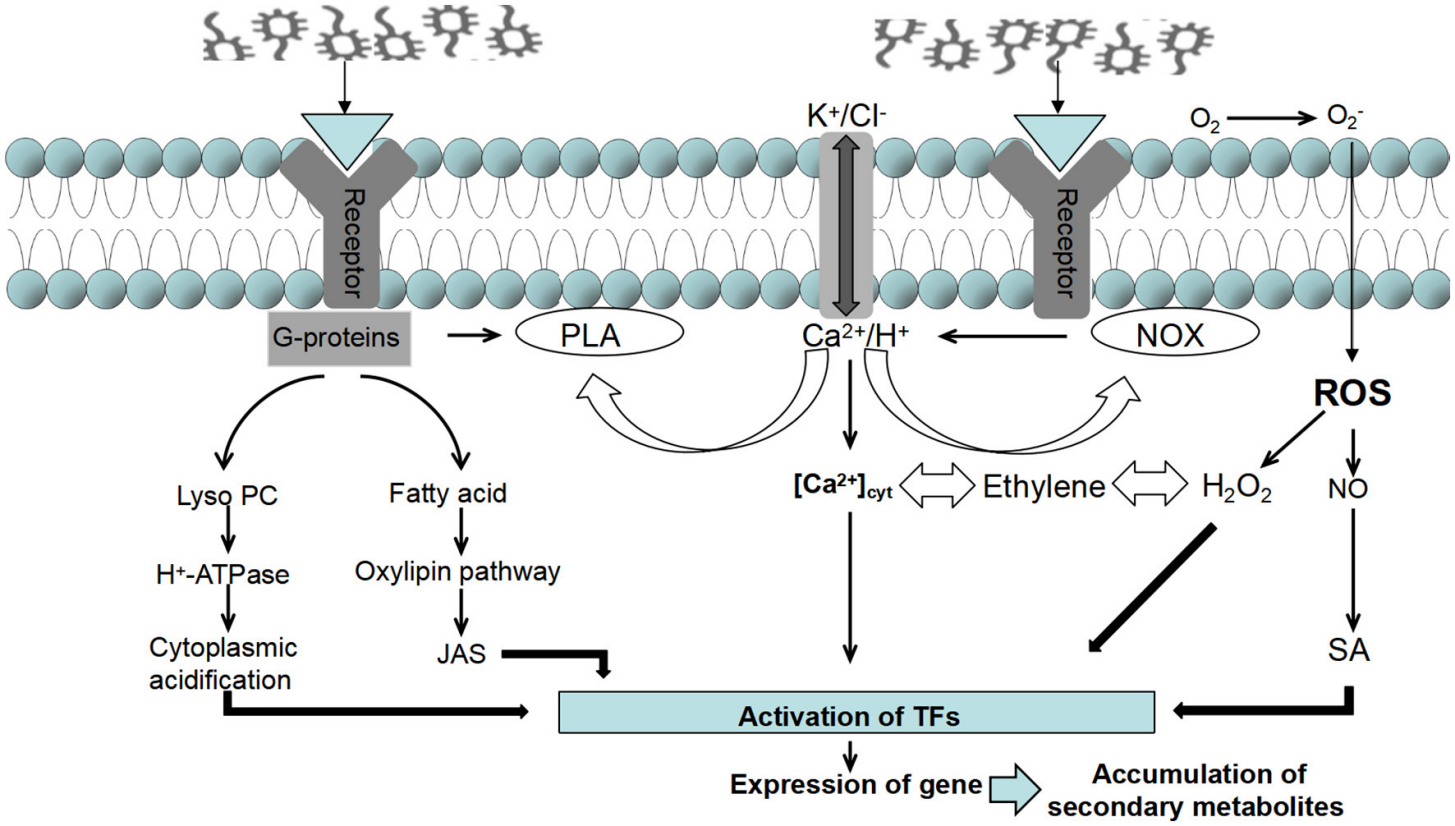
Signal transduction pathways of endophytic elicitors in medicinal plants.

This confirms the molecular mechanism of endophytic elicitors directly inducing the synthesis of volatile oil from *Atractylodes macrocephala* through the SA signal pathway. In summary, the signal molecules that induce the synthesis of plant secondary metabolites intersect, restrict, and coordinate with each other. This interplay occurs through complex signal networks and influences the expression of related genes, alters the activity of related enzymes, and mediates the synthesis and accumulation process of plant secondary metabolites (Figure 5).

### 3.3 Transcription factor integration and key gene expression

Transcription factors regulate the co-expression of multiple genes involved in production of secondary metabolites in medicinal plants by activating or inhibiting the transcription process. In the induction mechanism of most endophytic elicitors, signal molecules play a significant role in the secondary metabolism of host plants by bridging the indirect regulation of transcription factors by elicitors (Du et al., 2023). For example, Yang et al. reported that the rapid accumulation of MEJA in *Catharanthus roseus* cells after induction led to the integration of CrWEKY1, a member of the WRKY family in the metabolic pathway of terpene indole alkaloid precursors. This integration resulted in the overexpression of *TDC, STR*, and *D4H* genes, ultimately promoting the synthesis of alkaloid secondary metabolites (Yang et al., 2014). Similarly, under the influence of endogenous fungal elicitors, endophytic fungus NDZKDF13 enhances the gene expression of key enzyme LDC in the corresponding metabolic pathway, ultimately promoting the synthesis and accumulation of OMA in *Sophora flavescens* (Sun et al., 2018; Namdeo et al., 2002).

In addition, endogenous inducers can directly lead to the rapid accumulation of transcription factors in the AP2/ERF family within the cell, binding cis-elements, and thereby activating gene expression involved in the synthesis of alkaloids. This process ultimately promotes the accumulation of alkaloids (Yue et al., 2022). The relationship between transcription factors and various signaling molecules in the induction of plant secondary metabolites by medicinal plant endophytes remains poorly understood. Further research is necessary to enhance our understanding of the induction mechanisms of endophytes and to explore their potential for secondary metabolite production.

## 4 Application of endophytic elicitors in accumulation of secondary metabolites in medicinal plants

Medical plants are widely used as the main source of natural medicine in various fields. In the field of traditional medicine, such as Chinese medicine, Tibetan medicine, and Indian Buddhist medicine, medicinal plants provide primary healthcare services for approximately 80% of the population in developing countries worldwide (Aye et al., 2019). Furthermore, despite the rapid development of modern medicine a significant number of clinical drugs are still derived from natural products found in medicinal plants (Feng et al., 2024). Notably, some drug metabolites can now be directly obtained from their endophytic microbes and exhibit distinct pharmacological effects. Although endophyte-derived drugs are increasingly being applied at various clinical stages, their development and clinical adoption remain limited (Table 1). Accordingly, it is crucial to regulate the synthesis and accumulation of effective and active components in medicinal plants. In recent years, research on plant microbiology has deepened, leading to the gradual adoption of endophytic elicitors as an important method to enhance the production of secondary metabolites from medicinal plants (Cao X. et al., 2022). This mechanism has been successfully applied to the accumulation of effective active ingredients in various valuable traditional Chinese medicines. The newly synthesized pool of biologically active metabolites includes characteristic metabolites with medical and pharmaceutical potential, such as alkaloids, terpenes, flavonoids, and saponins (Table 2).

**Table 1:** Clinical research and development applications of endophytic drugs.

**Drug type**	**Drug name**	**Source of endophytic genera**	**Clinical outcomes**	**Clinical phase**	**References**
Anti-tumor effects	Paclitaxel	Fungal-ST026*(Altemaria alternate var.monosporus)*	Inhibits microtubule depolymerization and stabilizes microtubules	Post-market monitoring	(Cheng et al., 2011)
	Vincristine	*Fusarium oxysporum*	Treatment of Hodgkin's disease, acute and chronic lymphoblastic leukemia, malignant lymphoma, various cell tumors and breast cancer	Preclinical development	(Wei and Wang, 2020)
	Camptothecin	*Alternariaburnsii NCIM 1409*	Treatment of head and neck tumors, bladder cancer, leukemia	Preclinical studies	(Natarajan et al., 2023)
	Polysyllabic acid (GWT)	*Penicillium AL- ternaria*	Microtubule or DNA damaging ability with broad-spectrum and efficient anti-tumor effects	Preclinical studies	(Biswas et al., 2020)
Antioxidant action	Graphislactone A	*Cephalosporium sp*. IFB-E001	Removes DPPH and -OH more effectively than vitamin C and BHT	Preclinical studies	(Song et al., 2005)
Anti-cardiovascular diseases	Propranolol (beta-blocker used treat high blood pressure)	*Glomerella cingulata*	Beta-adrenergic receptor blocking drug that exclusively and efficiently converts propranolol (S-configuration) to 4-OHylated propranolol, resulting in large amounts of 4-OHylated propranolol	Preclinical development	(Borges et al., 2009)
Antibiotics	Dicerandrols	*Phytophthora (*genus of flowering plants*)*	Antifungal effect	Preclinical development	(Wagenaar and Clardy, 2001)
Anti-diabetic	L-783,281	*Pseudomassaria sp*.	Phosphorylation of the tyrosine subunit of the human insulin receptor and phosphorylation of the substrate for the action of the insulin receptor activates Akt and exerts an insulin-like mediating effect	Preclinical development	(Salituro and Pelaez, 2001)
Immunosuppressant active substance	Hypromellose	*Fusarum subgluti- nans sp*.	Immunosuppressive activity	Preclinical studies	(Zhou et al., 2022)
Synthetic nanoparticles	Green silver nanoparticles	*Aspergillus Aureoles*	Widely researched as a broad-spectrum antimicrobial agent capable of releasing Ag+ ions to disrupt bacterial membranes and interfering with DNA/RNA replication, thereby destroying the bacteria without creating resistant bacteria	Preclinical studies	(Dinesh et al., 2021)

**Table 2:** Effect of endophytic elicitors on the accumulation of secondary metabolites in medicinal plants.

**Product type**	**Endophytes**	**Inducer source**	**Medicinal plant**	**Inoculation time**	**Culture condition**	**Effect results**	**Reference**
Alkaloids	*F. solani Absidia cristata Fusarium moniliforme Sheld*	Mycelium homogenate	*Catharanthus roseus*	Day 7 of subculture	Optimum addition amount: 5–30 mg/l carbon hydrate equivalent Processing time: 3 days	Induce a 2–5-fold increase in the content of different types of indole alkaloids	Zhao et al., 2001
	*Manoectochila Mycena dendrobii Mycenaorchidicola*	Mycelia and metabolites	*Anoectochilus roxburghii*		Optimum addition amount: Ethyl acetate extract from 20% (V/V) mycelium and 10 mg/l fermentation broth	Promote metabolite accumulation and lateral bud proliferation	Gao and Guo, 2001
	*Trichoderma harzianum*	Fungl culture filtrate	*Vinca minor*		Optimum addition amount: 10% (V/V)	The growth index and total alkaloid content of hairy roots were 1.52 and 1.16 times higher than those of the blank group, respectively	Verma et al., 2014
Phenols	*Epichloë bromicola* SH09	Solid fungus fertilizer	*Salvia miltiorrhiza*	Day 20 of subculture	Salvia miltiorrhiza seedlings were co-cultured for 60–120 d	The accumulation of tanshinone and danshen phenolic acid was significantly increased	Wu et al., 2022
	*C.nigricolor 3-G7 B. spectabilis 1-N2*	Mycelium	Bletilla striata	Day 10 of subculture	Day 115 after inoculation culture	The total phenol content was increased by 25.41%	Chen et al., 2021a
Terpenes	*Aspergillus fumigatus Penicillium janthinellum Nigrospora sp*.	Inactivated thallus	*Nicotiana tabacum L*.		Optimum addition amount: MICs ≤ 8 μg/mL	Induction of the accumulation of piperidol and sesquiterpenoid antibacterial compounds	Chen et al., 2022
	*Diaporthe sp*.	Mycelium	*Betula platyphylla*	Day 8 of subculture	Polysaccharide concentration of 40 μg/l	The mRNA level of the key enzyme LUS that produces triterpenoids and its product betulin reached 18 and 2.5 times higher than that of the control within 24 h	Fan et al., 2013
	*A. niger Saccharomyces Ag.rhizogenes Bacillus subtilis Escherichia coli*	Cell-free extracts	*Gymnema sylvestre*	Start of standstill period (11^th^ day)	Optimum addition amount: 0.5%−2.0% (V/V)	Increase the content of gymnosporin acid	Bhuvaneswari et al., 2013
	*Gilmaniella sp*.	Mycelium	*Atractylodes lancea*	30 Day of subculture	Optimum addition amount: 0.15 mg carbohydrate equivalent Processing time: 10 days	Eucalyptus oil alcohol content increased to 4.07 times that of the control group	Wang et al., 2012
	*Fusarium spp*.	Fungal extract	*Euphorbia pekinensis*	21 Day of subculture	Cell suspension culture system	Increase the content of isooriginin and originol by 5.81 and 3.56 times as much as that of the control group, respectively	Gao et al., 2011
	*Fusarium mairei*	Extracellular polysaccharide	*Taxus cuspidate*		Long culture and high ventilation	Increased the production of paclitaxel by 4.7 times in the blank group	Li and Tao, 2009
	*Aspergillus niger*	Inactivated thallus	*Taxus cuspidate*	Late exponential growth	Optimum addition amount: 40 mg/l carbon hydrate equivalent	Induce more than twice the yield of paclitaxel	Wang et al., 2001
	*F.oxysporum*	Cell wall extract	*Taxus cuspidate*	The 18^th^ day at the end of exponential growth	Optimum addition amount: 60 μg/ml (measured as sugar)	Induce taxol synthesis up to 5 times that of the control	Zhang et al., 2001
	*Verticillium dahliae*	Inactivated thallus	*Artemisia annua Linn*.	End of exponential growth	Optimum addition amount: 0.4 mg sugar per ml of culture medium Processing time: 4 days	The accumulation of artemisinin in hair roots increased by 45% compared to the control	Wang et al., 2000
Flavonoids	*Bionectria pityrodes Fusarium oxysporum Alternaria sp*.	Mycelial polysaccharide	*Tartary Buckwheat*	16 h	Optimum addition amount: 1.0 g/l	Rutin yield reaches 1.5–1.6 times of the control	Zhao et al., 2014
	*Fusariumoxysporum f.sp. albedinis*	Mycelial wall extract	*Phoenix dactylifera*		Optimum addition amount: 10 mg mycelium per milliliter 121°C for 45 min	The activity of phenylalanine ammonia lyase increases rapidly	Modafar et al., 2001
	*Sphaeropsis sp. B301*	Mycelium	*Ginkgo biloba*	8 Day of subculture		Flavonoid content is 1.8 times that of the control group	Hao et al., 2010
Saponins	*Fusarium oxysporium Dzf17*	EPS, WPS, SMP	*Dioscorea zingiberensis*	25d	Optimum addition amount: 20 mg/L	The content and yield of diosgenin reached 1.34, 2.85, 3.83, and 8 times that of the blank group	Li et al., 2011
	*Fusarium oxysporium Dzf17*	Paraspirodinaphthalene compound	*Dioscorea zingiberensis*	25d	Optimum addition amount: 20 mg/L	The yield of diosgenin is about 8 times that of the blank group	Mou et al., 2015
Lignans	*Fusarium graminearum Trichoderma viride*	Inactivated thallus	*Linum album*		Optimum addition amount: 190 mu g g(−1) DW 260 mu g g(−1) DW 160 A mu g g(−1) DW	Wheat grain fungus increased the contents of podophyllotoxin and 6-methoxy podophyllotoxin by 2 and 3 times, respectively, compared to the control group; and green mold increased the content of 6-methoxy podophyllotoxin by 2.4 times compared to the control group	Bahabadi et al., 2014
Organic acids	P. *aphanidermatum*	Fungal extracts	*Daucus carota*	5d	Optimum addition amount: 20 ml cell suspension 26°C Avoid light	Induction of p-Hydroxybenzoic Acid Production	Schnitzler et al., 1992
	Mucor fragilis	Mycelium extract	*S. miltiorrhiza*	20 Day of subculture		SmMf elicitor has the potential to enhance phenolic acid and tanshinone accumulations	Xu et al., 2021
Coumarins	*P. aphanidermatum*	Fermentation culture filtrate	*Cichorium intybus L.cv.Lucknow*		Optimum addition amount: 1.0% (V/V)	Induction of Hairy Root Growth and Coumarin Synthesis	Bais et al., 2000
Quinones	*Botrytis cinerea*	Cell wall polysaccharide	*Rubia tinctorum*	7 Day of subculture		Promoting the synthesis of anthraquinones	(Orbán et al., 2007)

Endophytic elicitors have become an effective application tool in traditional Chinese medicine research and plant cell culture. For instance, endophytic bacteria obtained from the medicinal plant *Euphorbia geniculate* can produce alkaloids and terpenes with antibacterial activity (Ikram et al., 2020). Therefore, utilizing an endogenous inducer mechanism to regulate the synthesis of secondary metabolites not only enhances our understanding of the molecular mechanisms underlying plant-microbe interactions but also offers a sustainable solution to the shortage of natural drug resources. Additionally, it modernizes traditional Chinese medicine by establishing a model of green innovation.

## 5 Prospects

Endophytic fungi, an important biological resource, play a role in both plant growth and enhancing plant resistance in an environmentally friendly manner. However, the widespread application of industrial production of natural drugs using endophytes has not been fully realized yet, and there are still many problems to be solved in the development and application of endophytes. While the application prospects of endophytic bacteria producing secondary metabolites through improved strain methods are promising, there is still a gap between practical applications in large-scale industrial production. Therefore, future research should focus on further exploring the abundant resources of endophytics and their complex relationships with plants to establish them as a high-quality source of new plant metabolites.

Endophytic elicitors induce the synthesis of secondary metabolites in medicinal plants through various complex and diverse mechanisms. These mechanisms are strictly regulated by a range of factors within and outside the cell. Each link represents a specific expression pattern in regulation and metabolism that ultimately translates into a noticeable physiological response, resulting in an increase in secondary metabolites. When studying the regulatory mechanism of endophytic bacteria systematically, it is necessary to fully consider the influencing factors. These factors include the type, concentration, addition time, induction time, and culture system of endophytic elicitors. Additionally, it is necessary to analyze the functional characteristics and interrelationships of each essential element in the interaction process. These elements include specific receptors, signal molecules, transcription factors, key enzymes, and expression genes. Although current research on endophytics as inducers to increase the content of effective components in medicinal plants is still limited, it has been revealed that endophytics are the best source of stimulation for promoting the synthesis and accumulation of effective components in medicinal plants.

The complex interaction between endophytics and host plants means that plants are likely to trigger an innate immune response when recognizing endophytic elicitors (Hacquard et al., 2017). During an immune response, plants respond to this stress stimulus by producing antioxidants and inhibiting their own growth. However, currently, the key factors that enable host plants to successfully activate the induction mechanism during an immune response, the co-evolution mechanism that progressively formed during continuous co-symbiosis between endophytes and host plants, and the repayment mechanism in the evolutionary relationship still need to be explained. Numerous recent studies have confirmed that specific metabolites associated with endophytic genes can participate in plant immune processes through the production of plant hormones such as indoleacetic acid and gibberellin. This discovery provides a reasonable explanation for the coordinated relationship between the induction mechanism and immune response.

Most current research focuses on the interaction between a single endophyte and a plant. However, it is important recognize that in natural ecosystems, the regulation of host plants is commonly achieved through the microbiota of endophytes (Ku et al., 2024). Therefore, more research is needed to explain the assembly process of microbial communities and the impact of microbial communities on host plants. Understanding the interactions among various microbial groups, as well as within them, is a prerequisite for investigating the regulation of endophytic microbial communities. The composition and effectiveness of endophyte communities are controlled and determined by a number of drivers, including the host immune system, host genotype, environmental factors, microbial interactions, and soil and nutrient types. Furthermore, by creating artificially synthetic microbial communities that closely resemble the structural patterns of plant-microbe interactions, it may be possible to explore the intricate relationships between plant-microbe-environment modules through the gradual reconstruction and manipulation of microbial community members and environmental parameters (Chesneau et al., 2025). In order to create more effective synthetic microbial communities, the metabolic modeling of microflora can also be utilized to determine the metabolic activities of various combinations of microorganisms and validate their internal gene expression (Ruan et al., 2024). Therefore, based on the above strategies for constructing synthetic microbial communities, further in-depth investigation of the relationship between endophyte microbiota and host plants will help to accelerate the development and utilization of abundant endophyte resources.

The advancement and maturation of various technologies and disciplines, such as cell biology, molecular genetic methods, metagenomics, and bioinformatics, may provide a more comprehensive explanation of the diverse composition of endophytes in symbiotic environments, their life history, and their interactions with plants. We can extensively screen the diversity and community structure of endophytic microbiomes, design and use a new dual genome-based symbiosis chip tool to study the symbiosis between endophytic bacteria and their host plants (Tai et al., 2023), reveal and explore the physiological and ecological functions of endophytic elicitors in medicinal plants, and fully dissect the complete pathway for inducing events. This will continuously enrich the content of the human drug treasure house, bring strategic improvements, and strengthen the circular and sustainable development of medicinal plant active ingredients with medical and industrial value in medicine, food, agriculture, and forestry.
